# The relationship between HIV/AIDS and coronary heart disease: A bibliometric analysis

**DOI:** 10.1097/MD.0000000000039831

**Published:** 2024-10-04

**Authors:** Qiong Cai, Wei Pan, Chunming Zhang, Xianhui Zhang, Chunjie Wang, Yan Sun, Mingyang An, Fang Pan, Jiangping Xiao, Xilong Pan

**Affiliations:** a Department of Social Medicine and Health Education, School of Public Health, Peking University, Beijing, China; b The Sixth Peoples Hospital of Zhengzhou, Zhengzhou, China; c ZaiShuiYiFang Smart Technology Limited Company, Hubei, China.

**Keywords:** AIDS, bibliometric analysis, citeSpace, coronary heart disease, HIV, research hotspots

## Abstract

**Background:**

Acquired immunodeficiency syndrome is a malignant infectious disease caused by the human immunodeficiency virus (HIV). HIV gradually destroys the body’s immune system and weakens the body’s ability to resist diseases. People living with HIV may have a higher incidence of coronary heart disease than people without HIV.

**Method:**

A literature retrieval from January 1, 1993 to October 1, 2023 based on the Web of Science Core Collection database. CiteSpace6.2.R4, VOSviewer v1.6.19, and Microsoft Excel 2019 were utilized for analyzing the following terms: countries, institutions, authors, journals, references, and keywords.

**Results:**

There were 1144 articles. The highest number of articles is in the USA, followed by Italy. University of California System, Harvard University, and Johns Hopkins University were the top 3 most productive institutions with publications in this field of research. Journal of Infectious Diseases ranked first with the highest publications (532 records), followed by Immunology (362 records), and Cardiac Cardiovascular Systems (242 records). Keyword co-occurrence analysis showed antiretroviral therapy, myocardial infarction, and protease inhibitors, etc. Keyword cluster analysis obtained 13 categories, which were roughly divided into 3 themes: (1) cardiovascular disease that has occurred or may occur; (2) HIV acquisitions that have occurred; (3) risk factors for cardiovascular disease.

**Conclusion:**

The article obtained the hotspots and trends and provided references for subsequent research. Based on the keyword citation burst detection analysis, we speculated that heart failure, risk, subclinical atherosclerosis, infection, and association were the research hotspots in recent years, which had a certain predictive effect on the future research direction.

## 1. Introduction

Acquired immunodeficiency syndrome (AIDS) is a disease caused by human immunodeficiency virus (HIV) infection that severely impairs the function of the immune system. CD4^+^ T-lymphocyte count is the leading indicator of the stage of HIV infection and the effectiveness of treatment.^[1]^ Antiretroviral therapy (ART) is an effective method to inhibit HIV replication and reinvigorate the immune system.^[2]^ The Joint United Nations Programme on HIV/AIDS estimates that in 2022. In the world, 39 million people living with HIV (PLHIV) and 29.8 million PLHIV are on ART.^[3]^ Although ART effectively reduces the harm associated with HIV infection, it doubles the risk of cardiovascular disease in PLHIV.^[4–6]^ After receiving ART, PLHIV generally have vascular inflammation,^[7,8]^ insulin resistance,^[9]^ dyslipidemia,^[9,10]^ increased visceral adipose tissue,^[11]^ and immune activation,^[6]^ which will increase the risk of coronary heart disease (CHD). Compared with people without HIV, PLHIV are more likely to have heart failure^[12]^ and have higher intramyocardial triglyceride content.^[13]^ Intramyocardial triglyceride content is one of the significant factors contributing to the increased risk of CHD.

However, after searching various popular databases, we found considerable literature on the relationship between the risk of HIV/AIDS and CHD. Although some scholars have done literature review studies,^[14–16]^ the studies’ systematization and comprehensive visual analysis still need to be improved.

Bibliometric analysis studies the relationship between scientific literature and citation. It evaluates the relationship between a scientific research output, influence and academic cooperation through mathematical, statistical methods and provides a scientific decision-making basis through data analysis. Bibliometric analysis studies help researchers understand the dynamics of the academic community and guide research direction and resource allocation.^[17]^ CiteSpace software is a visual tool developed by Chaomei Chen, a Chinese-American from the School of Computing and Information at Drexel University,^[18]^ which can extract information from keywords and produce cluster labels according to Log-likelihood rate, Latent Semantic Indexing, and Mutual information algorithms. The latest version is CiteSpace 6.2.R4. VOSviewer software is a bibliometric and analysis software developed by the Centre for Science and Technology Studies of Leiden University in the Netherlands,^[19]^ which draws scientific knowledge maps to visually show the knowledge structure. The latest version is VOSviewer v1.6.19. Both tools play a very important role in the literature research of specific fields and can analyze and predict the research hotspots and development trends in related fields.^[20]^

Web of Science Core Collection (WoSCC) database is an important database for obtaining global academic information. It contains all articles and citations from the vast majority of journals, ensuring the integrity and systematicity of research in terms of time and content. Therefore, this study intended to use CiteSpace to visually analyze the literature related to the relationship between HIV/AIDS and CHD in the WoSCC database to explore the main research content and track the research hotspots. Based on the data analysis findings in the literature, we analyze the development dynamics and research trends of HIV/AIDS and CHD correlation research in the international arena, in order to provide a reference for predicting the direction of further research on the relationship between HIV/AIDS and CHD and to promote the development of effective strategies for preventing and treating CHD in PLHIV.

## 2. Methods

### 2.1. Data source and search strategy

Our study used the most common and popular database for bibliometrics, the WoSCC database(https://www.webofscience.com/wos/woscc/basic-search), to retrieve literature. The retrieval method was topic searching including searching title, abstract, author keywords, and keywords plus. Using keywords related to AIDS, including “human immunodeficiency virus,” “HIV,” and “AIDS”; and CHD-related keywords, including “coronary heart disease,” “CHD,” and “coronary diseases,” and operation method was “AND.” All obtained records results were downloaded in plain text file format for subsequent analysis. The first published paper traced back to 1993, proposed by Segal, BH.^[21]^ We attempted to find all relevant studies in the whole field, and therefore we selected our research period from 1993 to 2023. Then, we completed all searches and data exports on the same day (October 1, 2023) since metrics constantly changed.

### 2.2. Inclusion and exclusion criteria

The inclusion and exclusion of studies were based on the filters of the WoSCC database. The studies that met the following criteria included: (1) Articles published in the period from January 1, 1993, to October 1, 2023; (2) Articles about HIV/AIDS and CHD; (3) Original articles; (4) Published in English. Exclusion criteria were: (1) Duplicate publications; (2) Proceeding paper, editorial material, meeting abstract, book chapters, letters, early access, correction, reprint, and other types.

### 2.3. Data collection and cleaning

The following basic information was collected for each article: countries, institutions, journals, authors, references, and keywords. Keywords with the same meaning but in different styles were standardized: “coronary-artery disease” was replaced by “coronary heart disease,” and “human immunodeficiency viru” was replaced by “human immunodeficiency virus.” Two researchers strictly screened the literature by examining the abstract section according to the inclusion and exclusion criteria. The third researcher determined the contents with differences and uncertainties. All documents were imported into EndNote X9 for document de-duplication and management.

### 2.4. Bibliometric software

The above plain text files were imported into CiteSpace 6.2.R4 software and VOSviewer v1.6.19 software. CiteSpace is an academic literature analysis tool with an advanced visualization feature, which has the advantage of in-depth mining of academic research. It is able to extract burst terms and reveal the hotspots, developments trends and evolutionary paths of academic research. Compared with CiteSpace, VOSviewer more intuitively displays the relationship between network data and realizes the purpose of speedily gaining a comprehensive picture of research topics.

### 2.5. Data analysis

In the knowledge map generated by CiteSpace, research projects were presented, including countries, institutions, authors, keywords, etc. VOSviewer mapped the scientific network of co-citation analysis. Each research project represents a node. The larger the node, the larger the number of papers published, the number of citations, the frequency of occurrence, and other related indicators. The connection between the nodes represents the relationship of cooperation, co-occurrence, and citation. The network map analysis demonstrates the nodes’ research level and capabilities and shows the cooperative relationship between each node. The timezone shows research trends based on the network map in the form of a timeline. Reference co-citation cluster analysis reflected and summarized the main content of the research field. The research themes of HIV/AIDS and CHD are interpreted by co-occurrence analysis of keywords, cluster analysis of Ridgeline Plot, and emergence analysis over the past 30 years. The trend of literature publication in the past 30 years was plotted using Microsoft Excel 2019.

### 2.6. Research ethics

All data were available on a public database. Therefore, permission was not required from the ethics committee.

## 3. Results

### 3.1. Search results

**Figure 1.**
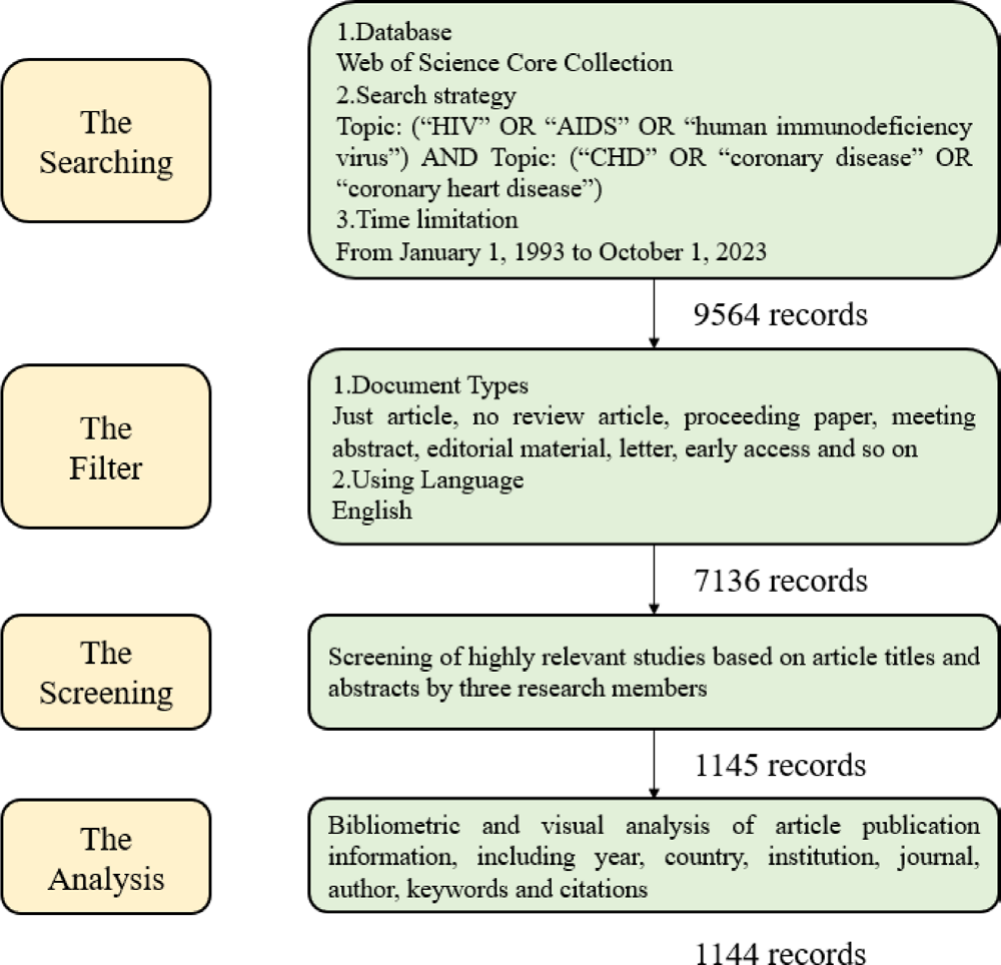
A searching flow diagram.

**Figure 2.**
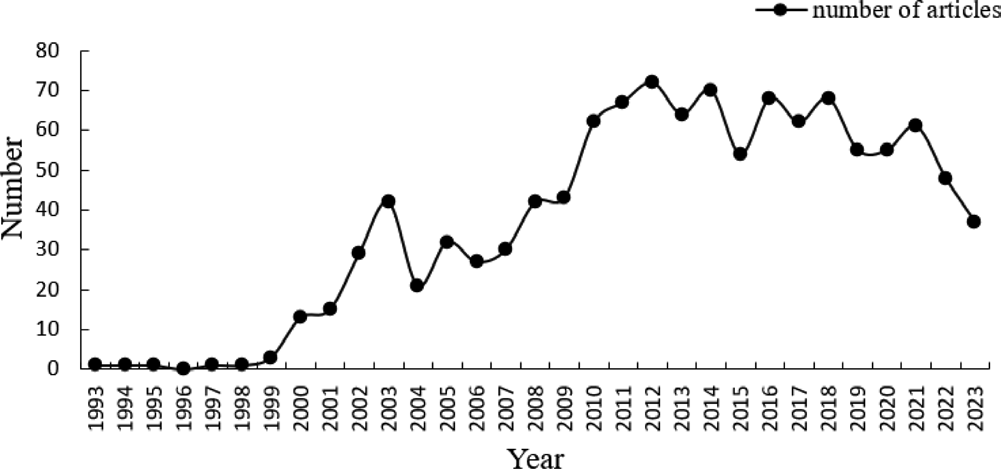
The distribution trend chart from January 1, 1993, to October 1, 2023.

From January 1, 1993, to October 1, 2023, the final search results had 1144 literature records, and the search process is shown in Figure 1. Fundamentally, all the articles are original articles, which greatly reflect the development trends and changes in the field of research on the association between HIV/AIDS and CHD. The number of articles published continued to increase during the early phase of the study, peaking in 2012 (n = 72). Between 2012 and 2023, the number of publications will remain stable and fluctuate up and down (Fig. 2), and research output is expected to continue to increase in 2024.

### 3.2. Analysis of author’s country of origin

**Figure 3.**
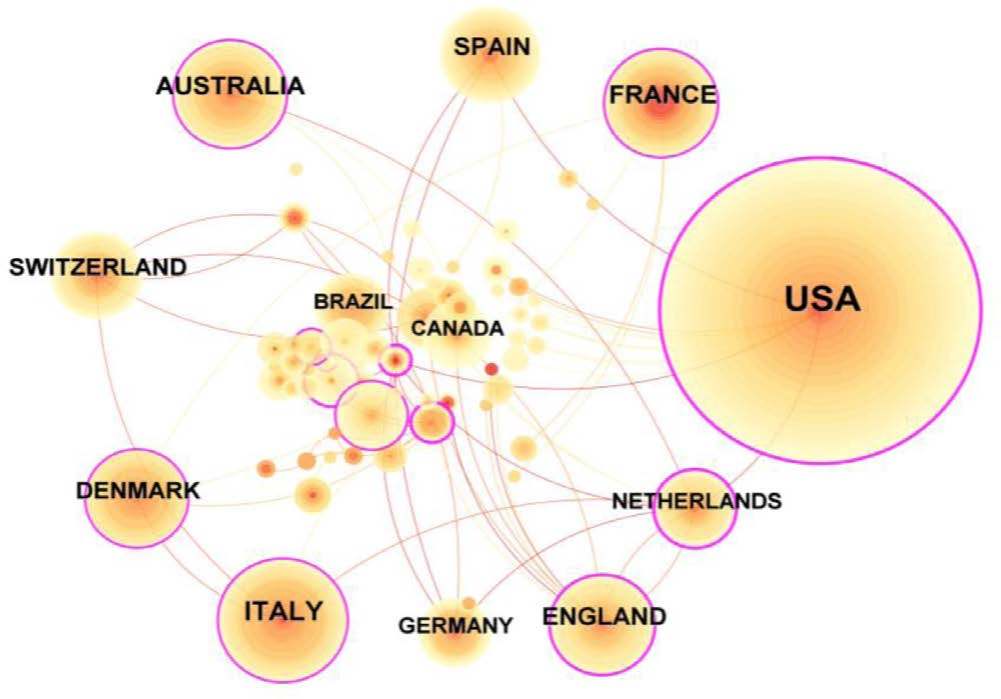
Mapping of country cooperation networks.

In CiteSpace software, the Node Type is “Country,” with 1144 articles from 70 countries. The mapping of the country’s cooperation network is shown in Figure 3. Table 1 lists the top 10 countries in terms of the number of articles, and the highest number of articles is the United States of America (n = 648), followed by Italy (n = 118), France (n = 84), Australia (n = 84), and United Kingdom (n = 76). The United States is the leading country in the study of the association between HIV/AIDS and CHD, with more collaboration with other countries around the world. Then, VOSviewer v1.6.19 was used for citation analysis. There were 33,569 citations in the United States, followed by Australia (9058 citations), and Denmark (8582 citations). However, in terms of the average number of citations per paper, Denmark was the highest (122.6 citations/paper), followed by Switzerland (109.01 citations/paper), and Australia (107.83 citations/paper).

**Table 1:** Ranking of top10 country of articles numbers.

Rank	Country	Number	Percent	Citations	Average citations
1	United States (USA)	648	37.94%	33,569	51.80
2	Italy	118	6.91%	6287	53.28
3	France	84	4.92%	6670	79.40
4	Australia	84	4.92%	9058	107.83
5	United Kingdom (England)	76	4.45%	6923	91.09
6	Spain	75	4.39%	3066	40.88
7	Denmark	70	4.10%	8582	122.6
8	Switzerland	67	3.92%	7304	109.01
9	Netherlands	48	2.81%	4492	93.58
10	Germany	47	2.75%	1501	31.94

### 3.3. Analysis of universities and institutions

From January 1, 1993, to October 1, 2023, a total of 988 institutions were involved in research on the association between HIV/AIDS and CHD. The number of papers published by scientific research institutions can represent their research ability and research level in this field. The top 15 universities or institutions in the number of publications are almost all in the United States, as shown in Table 2. The University of California System (USA) is the institution with the highest number of publications, followed by Harvard University (USA), and then Johns Hopkins University (USA). It was not until UDICE-French Research Universities (France), ranked 13, that the first country other than America appeared. The top 15 institutions published 1233 articles, accounting for 42.39% of the total.

**Table 2:** Ranking of top15 universities and institutions of article numbers.

Rank	Universities and institutions	Number	Year	Centrality
1	University of California System	168	1994	0.01
2	Harvard University	130	1995	0.06
3	Johns Hopkins University	106	2003	0.07
4	University of California Los Angeles	92	1994	0.20
5	Pennsylvania Commonwealth System of Higher Education (PCSHE)	86	2007	0.02
6	Massachusetts General Hospital	81	2005	0.05
7	Harvard Medical School	80	1998	0.03
8	University of California San Francisco	73	2004	0.05
9	University of California Los Angeles Medical Center	68	1994	0
10	Northwestern University	65	2008	0.06
11	University of Pittsburgh	64	2008	0.08
12	Johns Hopkins Bloomberg School of Public Health	60	2003	0.11
13	UDICE-French Research Universities	55	2000	0.07
14	US Department of Veterans Affairs	54	2005	0.07
15	Veterans Health Administration (VHA)	51	2005	0

### 3.4. Analysis of author cooperation

**Figure 4.**
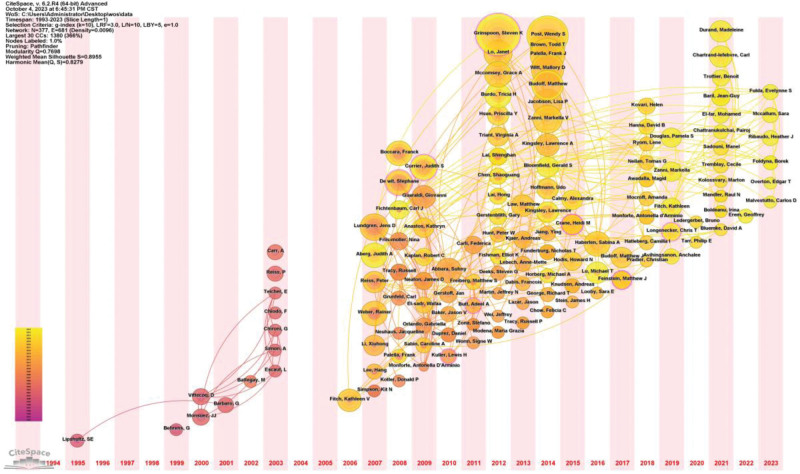
Author’s timezone map.

We obtained 377 authors while selecting “Author” for the parameter of Node Types and set the G-index to 10. When selecting Authors with “a count ≥3,” which represents the number of published papers more than 3, we obtained a total of 134 authors. According to the number of publications by the author and the cooperative relationship between the author and other authors, the author’s timezone map is drawn with time as the main line, as shown in Figure 4. Each node represents an author. The larger the circle, the more articles the author has published. The more connections there are, the more cooperative relationships the author has. Grinspoon, Steven K is the most productive author and has published 40 articles since 2012. Followed by Post Wendy S (n = 36), Brown Todd T (n = 26), Palella Frank J (n = 24), Budoff Matthew (n = 20), and Witt Mallory D (n = 20). A few authors, such as Carr A, had no contact or collaborative relationship with other authors.

### 3.5. Analysis of publications and journals

Through the statistics of relevant research publishing institutions from 1993 to 2023, a total of 65 journals were included in the study, and the list of the top 10 journals with the number of publications is shown in Table 3. In the past 30 years, AIDS (n = 110) published the most significant number of articles, followed by the Journal of Acquired Immune Deficiency Syndromes (n = 74). Clinical Infectious Diseases (n = 44) and HIV Medicine (n = 44) tied for the highest number of articles published at 44.

**Table 3:** Ranking of top10 publications and journals of article numbers.

Rank	Journals	Documents	Percent
1	AIDS	110	9.68%
2	Journal of Acquired Immune Deficiency Syndromes	74	6.51%
3	Clinical Infectious Diseases	44	3.87%
4	HIV Medicine	44	3.87%
5	PLOS One	36	3.17%
6	AIDS Research and Human Retroviruses	32	2.81%
7	Atherosclerosis	26	2.29%
8	Antiviral Therapy	22	1.94%
9	BMC Infectious Diseases	22	1.94%
10	The Journal of Infectious Diseases	22	1.94%

In addition, among the 1144 literature records, the ranking of the top 10 cited articles is shown in Table 4. Among the top 10 articles, the team led by Friis-Moller, N accounted for 3 papers, followed by Kuller, LH team with 1242 citations, and Triant, VA team with 1179 citations. Among them, in 2003, Friis-Moller, N published in the New England Journal of Medicine with 1293 citations, concluded that PLHIV have a relatively increased incidence of myocardial infarction during the first 4 to 6 years of combination ART.^[22]^

**Table 4:** Ranking of top10 citation articles.

Rank	Authors	Citations	Title	Journals	Institution
1	Friis-Moller, N (2003, Denmark)	1293	Combination antiretroviral therapy and the risk of myocardial infarction	New England Journal of Medicine	Odense University Hospital
2	Kuller, LH (2008, USA)	1242	Inflammatory and coagulation biomarkers and mortality in patients with HIV infection	Plos Medicine	University of Pittsburgh
3	Triant, VA (2007, USA)	1179	Increased acute myocardial infarction rates and cardiovascular risk factors among patients with human immunodeficiency virus disease	Journal of Clinical Endocrinology & Metabolism	Harvard University
4	Friis-Moller (2003, Denmark)	1130	Class of antiretroviral drugs and the risk of myocardial infarction	New England Journal of Medicine	Odense University Hospital
5	Freiberg, MS (2013, USA)	937	HIV infection and the risk of acute myocardial infarction	Jama Internal Medicine	University Pittsburgh
6	Friis-Moller (2003, Denmark)	809	Cardiovascular disease risk factors in HIV patients: association with antiretroviral therapy: Results from the DAD study	AIDS	Odense University Hospital
7	Grinspoon, S(2005, USA)	799	Medical progress - Cardiovascular risk and body-fat abnormalities in PLHIV.	New England Journal of Medicine	Massachusetts General Hospital
8	Sabin, CA (2008, US)	679	Use of nucleoside reverse transcriptase inhibitors and risk of myocardial infarction in PLHIV enrolled in the D:A:D study: A multi-cohort collaboration	LANCET	University College London
9	Neuhaus, J (2010, USA)	652	Markers of inflammation, coagulation, and renal function are elevated in adults with HIV infection	Journal of Infectious Diseases	University of Minnesota Twin Cities
10	Hadigan, C (2001, USA)	531	Metabolic abnormalities and cardiovascular disease risk factors in adults with human immunodeficiency virus infection and lipodystrophy	Clinical Infectious Diseases	NIH & NIAID

### 3.6. Analysis of reference co-citation

**Figure 5.**
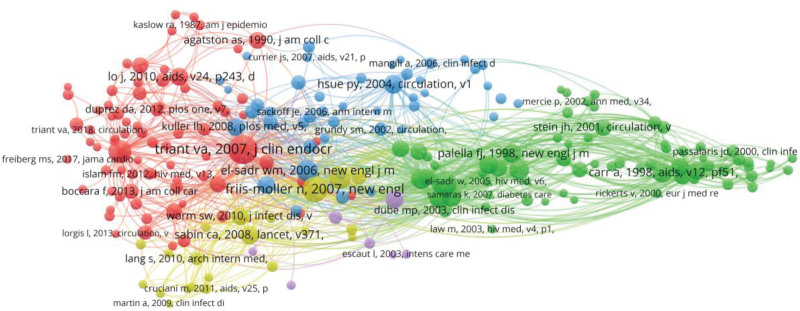
Network map of reference co-citation.

Co-citation analysis of cited documents of related research was conducted in VOSviewer software, and a total of 23,676 cited literature was obtained. Setting the Minimum number of citations of a cited reference to 20 times, the map of co-cited documents with 218 thresholds is obtained in the VOSviewer, as shown in Figure 5. The software classifies the above references into 5 categories, and a different color represents each category. Each node represents a cited document, and the node’s size indicates the number of citations. The larger the node, the higher the number of sources. The red nodes represent cluster #1, which contains 76 literature; The green nodes represent cluster #2, which includes 68 literature; The blue nodes represent cluster #3, which has 46 literature; The yellow nodes represent cluster #4, which contains 21 articles; The purple nodes represent cluster #5, which includes 7 articles. Triant Va published the highest of all the references in 2007. They published Increased Acute Myocardial Infarction Rates and Cardiovascular Risk Factors among Patients with Human immunodeficiency virus disease. The co-citation reached 308 times, and it was concluded that the risk of myocardial infarction and the risk of cardiovascular disease-related factors were higher in PLHIV, especially in women.^[23]^

### 3.7. Analysis of keyword co-occurrence

**Figure 6.**
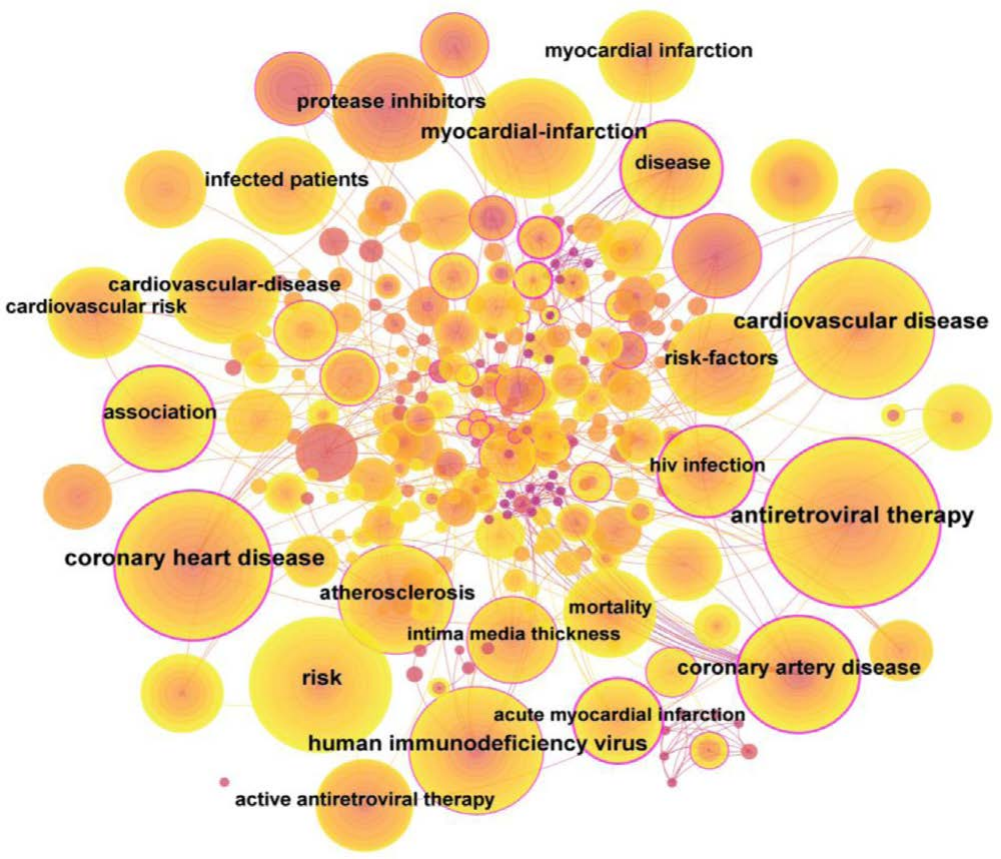
Keyword co-occurrence network mapping.

The CiteSpace software extracted 290 keywords when selecting Node Types “Keyword,” and setting the G-index to 10. Based on the frequency of keywords, the network map is drawn, which is shown in Figure 6. After excluding the keywords related to the search terms in the search strategy, such as “coronary heart disease” and “human immunodeficiency virus,” the top 15 keywords ranked by frequency of occurrence are detailed in Table 5. This shows that the popular directions in research on HIV/AIDS and CHD in recent years are antiretroviral therapy, myocardial infarction, risk factors, atherosclerosis, protease inhibitors, and association, etc.

**Table 5:** Ranking of top15 keywords usage frequency.

Number	Keywords	Frequency	Centrality
1	Antiretroviral therapy	371	0.11
2	Myocardial infarction	324	0.07
3	Risk factors	203	0.34
4	Atherosclerosis	158	0.07
5	Protease inhibitors	153	0
6	Association	138	0.15
7	Intima media thickness	135	0.18
8	Insulin resistance	120	0.05
9	Cardiovascular risk	115	0.12
10	Inflammation	77	0.01
11	C-reactive protein	73	0.09
12	Lipodystrophy	66	0.09
13	Infection	60	0.04
14	Immune activation	56	0.01
15	Metabolic syndrome	55	0

### 3.8. Analysis of keyword cluster

**Figure 7.**
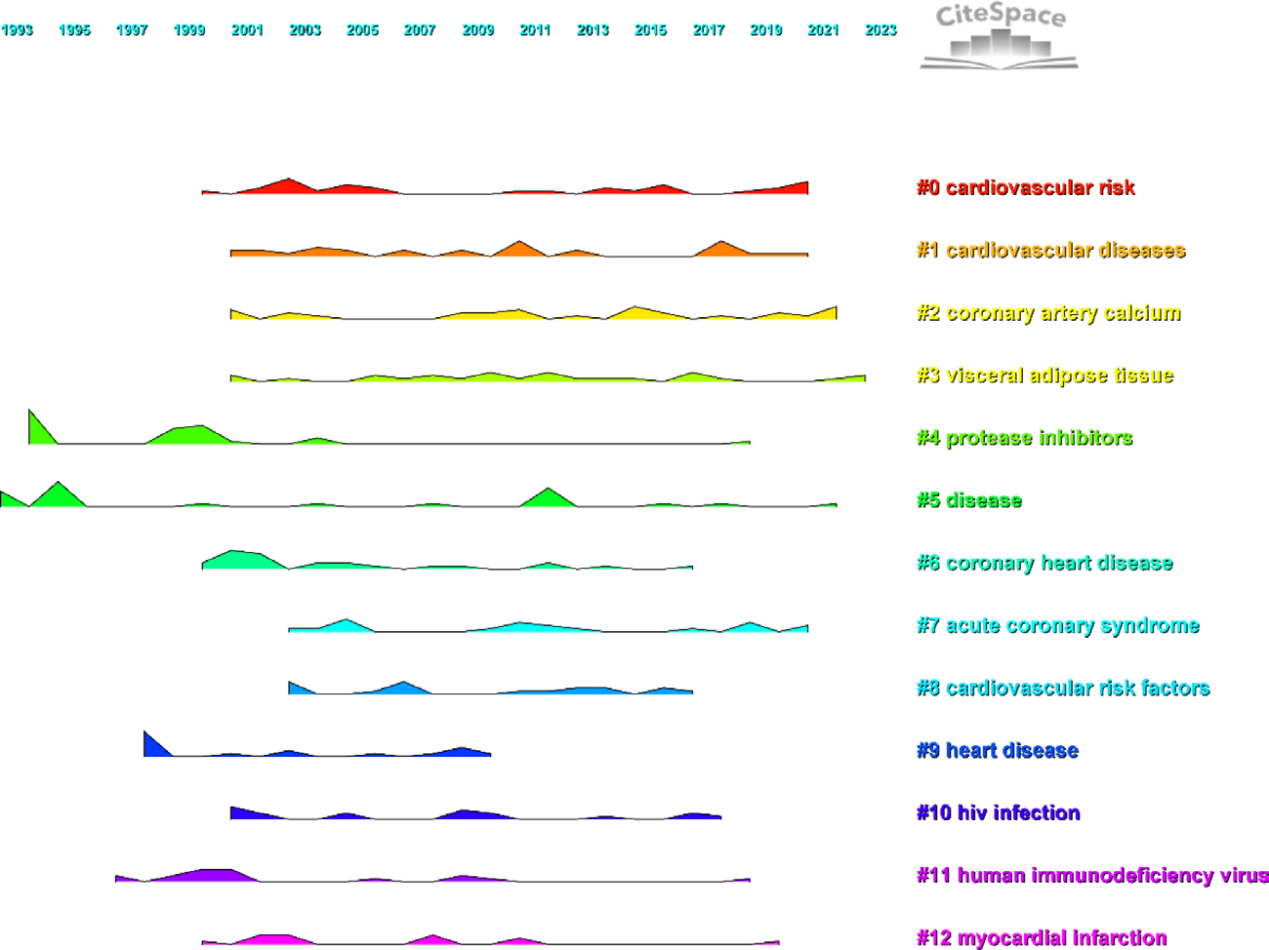
Ridgeline Plot for keyword cluster analysis.

In the cluster analysis, the Log-likelihood ratio algorithm is used to divide keywords into 13 categories according to different colors. For each category, the most representative keywords are selected by the algorithm as highly generalized labels. Through using the Ridgeline Plot, the changes in keywords over time are shown in Figure 7. As the spectrum parameters are Modularity Q = 0.77 > 0.3, Weighted Mean Silhouette S = 0.90 > 0.7, Harmonic Mean (Q, S value) = 0.83 > 0.5, it indicates that the clustering results have good credibility.^[24]^ By summarizing the clustering results, the main focus is based on 3 categories of topics: the first one is occurred or may occur cardiovascular diseases: #1 cardiovascular diseases, #6 coronary heart disease, #7 acute coronary syndrome, #9 heart disease, #12 myocardial infarction; The second category is acquired HIV: #5 disease, #10 HIV infection, #11 human immunodeficiency virus; risk factors for the third category of cardiovascular disease: #0 cardiovascular risk, #2 coronary artery calcium, #3 visceral adipose tissue, #4 protease inhibitors, #8 cardiovascular risk factors. The third category #4 protease inhibitors keyword was first proposed. Although few people have studied it since 2005, it lasted for a long time. Keyword #3 visceral adipose tissue was proposed relatively late, but it continues to the present, therefore, it has a possibility to be the main research direction in the future.

### 3.9. Analysis of burst detection

**Figure 8.**
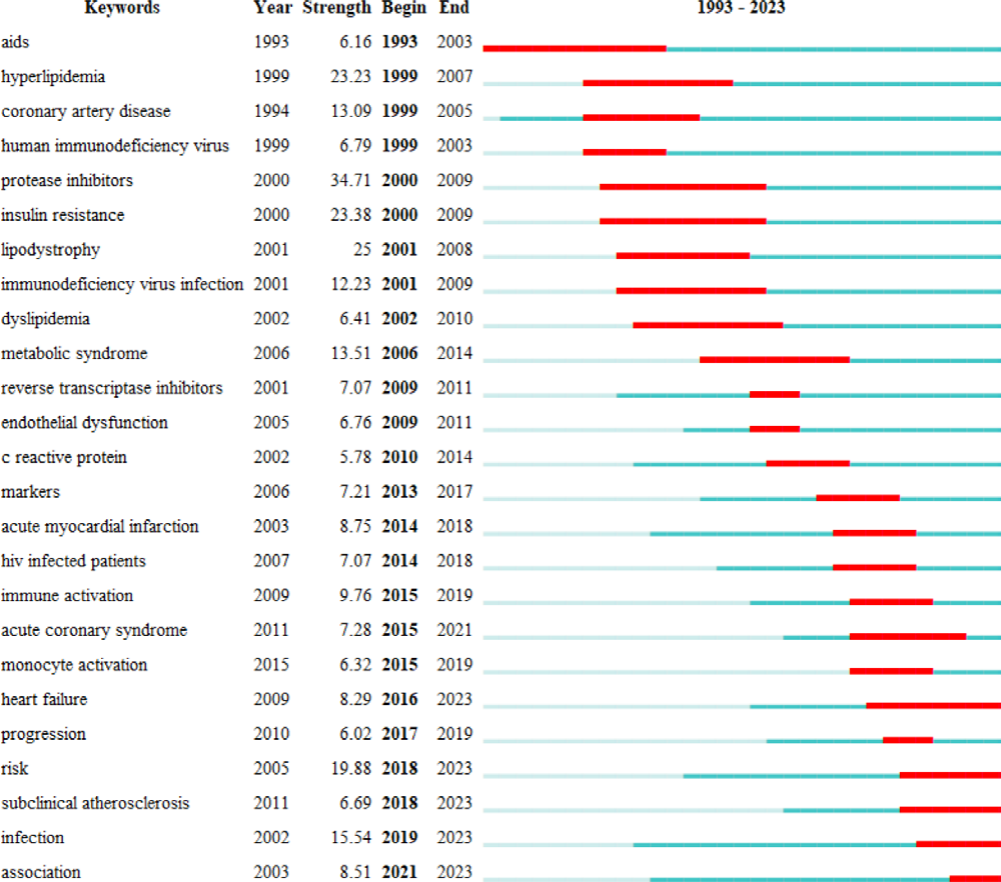
Top 25 keywords with the strongest citation bursts (“Year” represents the time of the first appearance of the keyword, “Strength” represents the intensity of the emergence, “Begin” represents the start time of the emergence, “End” represents the End time of the emergence, and the length between “begin–end” is equal to the length of the red area in the figure) aids acquired immunodeficiency syndrome.

On the basis of 3.8, we perform the burst detection analysis of keywords. Figure 8 shows the top 25 emergent words in the research field of the association between HIV/AIDS and CHD from 1993 to 2023. The keyword with the greatest burst strength is protease inhibitors (Strength = 34.71), followed by insulin resistance (Strength = 23.38). The keyword with the longest burst time is AIDS (Year = 10), followed by protease inhibitors (Year = 9) and insulin resistance (Year = 9). Since heart failure, risk, subclinical atherosclerosis, infection, and association have continued from its burst to the present, this may be the most popular keyword in the next few years.

## 4. Discussion

This study conducts a literature search and statistical analysis on the WoSCC database on the correlation between HIV/AIDS and CHD. A total of 1144 relevant literature were obtained, to a certain extent, they reflect the research trends and hotspots in HIV/AIDS and CHD. This research field primarily focuses on the cardiovascular diseases that have occurred or may occur in PLHIV, the existing late-stage HIV infection, and the influencing factors of cardiovascular diseases, etc, which indicates that this research field is popular in the era of CHD. According to the distribution map of the 30-year publication trend, the number of papers published increased rapidly from 1993 and peaked in 2012. The number of papers published fluctuated up and down around a stable value between 2012 and 2023. Accompanying the increase in ART coverage and the decline in mortality from HIV-related illnesses, non-HIV-related illnesses, mainly CHD, have gradually become the leading cause of death among PLHIV.^[25]^ Studies have shown that many of the mechanisms underlying the correlation between HIV/AIDS and CHD are still unclear,^[26]^ so the present study suggests that further exploring and analyzing the mechanisms of PLHIV CHD could help reduce the risk of death in PLHIV.

The number of publications and citations of research results reflect the scientific research strength and depth of different countries, institutions and universities. In terms of the number of publications, the United States has an absolute advantage. Ranking each research institution and university according to the number of publications, the University of California System has the largest number of publications, followed by Harvard University and Johns Hopkins University. As the top 10 research institutions and universities are all from the United States, it indicates that the United States has far more research investment in this field than other countries. The scientific research strength and economic capacity of the United States are relatively strong. In terms of the average citations of the articles, Denmark is the most prominent, which may be related to the 3 highly cited articles by the author Friis-Moller, N.^[22,27,28]^ Although many countries and institutions are cooperating and exchanging ideas, the cross-regional cooperation work can be done more tightly. In particular, institutional cooperation research within a country can promote coordination and resource sharing at the national level.

The timezone map is drawn with the authors’ appearance time and published papers, hence it can reflect the changing trend of the research field. Mapping based on the timing of the first publication and the number of publications by the authors revealed a small break in 2004, which is consistent with our publication trend map. Around 2012, the first appearance rate of authors in this field exploded, which indicating that they laid a solid foundation for the development of research in the next 10 years. Grinspoon, Steven K and his team have the greatest number of publications. They suggest that PLHIV on long-term combination antiretroviral therapy, especially protease inhibitors, have an increased incidence of myocardial infarction, even if treatment of PLHIV is more important than the risk of myocardial infarction. Friis-Moller, N and his group have the highest time cited. Among the first 10 articles, the Friis-Moller, N team occupied 3 articles, namely the first article,^[22]^ the fourth article,^[27]^ and the sixth article.^[28]^ They were followed by Kuller, LH team,^[29]^ Triant, VA team,^[23]^ and Freiberg, MS team.^[30]^ The top 5 frequently co-cited teams are Triant, VA team,^[23]^ Friis-Moller, N team,^[22,27]^ Klein, D team,^[31]^ Currier, and JS team.^[32]^ Among the journals published, the top 3 journals in terms of the number of articles were AIDS (n = 110), Journal of Acquired Immune Deficiency Syndromes (n = 74), Clinical Infectious Diseases (n = 44), and HIV Medicine (n = 44), with the latter 2 tied.

Keywords reflect the core research content of academic papers, and high-frequency keywords can reflect the research hotspots and trend evolution in this field. Therefore, keyword analysis is the most essential part of literature analysis. Keyword co-occurrence analysis was used to analyze the hot research directions in the field of HIV/AIDS and CHD in the past few years and reflected the current or future research hotspots and trends. By combining with the results of keyword burst detection analysis, it is reasonable to infer that the current research hotspots are heart failure, risk factors, subclinical atherosclerosis, and association. Heart failure is the most serious manifestation of CHD. An increasing number of studies have proved that HIV is an important factor in expanding the incidence of heart failure,^[33,34]^ and the association needs to be further studied. The majority of PLHIV may have subclinical cardiac dysfunction but lack of awareness. In 2030, nearly 80% of PLHIV are forecasted to have subclinical atherosclerosis.^[35]^ Since 2018, subclinical atherosclerosis has received high attention, and key research is conducive to the prevention, diagnosis, and treatment of the disease. Risk factors for CHD infection in PLHIV and their association have been the focus topics in these years. In the burst term list, they appear with high frequency and great strength. The increased prevalence of CHD in PLHIV should be attributed to HIV-specific factors such as vascular inflammation, traditional CHD risk factors such as obesity, and ART such as protease inhibitors,^[15,27,36]^ which is the result of the interaction of multiple factors.^[35]^ Research and analysis of the causes of PLHIV suffering from CHD again and actively taking measures to control the risk factors of concurrent CHD may be the future research needs and directions.^[37]^

In addition, through the cluster analysis of keywords, we obtained 13 categories. This study generalizes and summarizes the clustering results, which can be divided into 3 themes. The first theme is cardiovascular disease that has arisen or may occur. PLHIV have a higher risk of these diseases than people without HIV. Table 6 shows the representative keywords of each cluster after cluster analysis. The keywords are viewed according to the categories of the corresponding topics in the table, mainly focusing on the relationship between ART and CHD and other diseases. In recent years, an increasing amount of studies have proved that receiving ART is the cause of increasing the risk of CHD and other diseases. Focus on secondary branches that need attention. These could be cardiovascular diseases, CHD, coronary artery disease, acute coronary syndrome, heart disease, myocardial infarction, etc. The second theme is the current epidemic of HIV. ART prolongs the survival time of PLHIV and transforms the disease from high mortality to chronic disease.^[38]^ However, the effects of HIV and the secondary effects of long-term ART often carry the risk of heart failure and diastolic dysfunction. The third theme was risk factors for cardiovascular disease. HIV infection is associated with the up-regulation of inflammatory factors.^[39]^ Inflammation plays a significant role in cardiovascular diseases, and the migration and increase of inflammatory cells promote the formation of atherosclerosis.^[40,41]^ HIV can also cause endothelial dysfunction and immune activation, which can lead to an increased risk of CHD in PLHIV.^[42]^ Protease inhibitors and highly active antiretroviral therapy are common methods of ART, which play an effective role in the treatment of PLHIV. However, protease inhibitors potentially cause abnormal glucose and lipid metabolism in humans. Hyperlipemia and insulin resistance are risk factors for CHD.^[43]^ Highly active antiretroviral therapy predisposes to lipodystrophy.^[44,45]^ It causes visceral adipose tissue, insulin resistance and dyslipidemia.^[46,47]^ Although the benefits of ART treatment to PLHIV exceed the risk of inducing CHD, further research is needed to prevent the occurrence of heart disease and reduce unnecessary losses.

**Table 6:** Cluster analysis of keywords and top 5 keywords in the 13 clusters.

Cluster	Node	Keyword
#0	29	Cardiovascular risk (33.36), endothelial function (28.49), inflammation (26.11), activation (14.22), endothelial dysfunction (12.57).
#1	29	Cardiovascular diseases (23.08), infected patients (21.86), active antiretroviral therapy (17.94), blood pressure (10.87), oxidative stress (10.52).
#2	28	Coronary artery calcium (38.34), cardiovascular disease (22.09), atherosclerosis (16.52), immune activation (14.95), intima media thickness (14.23).
#3	26	Visceral adipose tissue (10.84), association (10.31), people living with HIV (9.81), insulin resistance (8.91), lipodystrophy (7.66).
#4	26	Protease inhibitors (39.29), insulin resistance (36.35), hyperlipidemia (28.97), coronary artery disease (26.78), lipodystrophy (21.85).
#5	25	Disease (30.11), therapy (23.17), outcome (17.51), acquired immunodeficiency syndrome (16.84), rates (14.14).
#6	24	Coronary heart disease (42.96), antiretroviral therapy (39.81), coronary artery disease (24.16), prediction (19.25), metabolic syndrome (12.47).
#7	19	Acute coronary syndrome (54.13), risk (20.6), acute myocardial infarction (19.08), percutaneous coronary intervention (10.26), pharmacokinetics (9.72).
#8	18	Cardiovascular risk factors (11.96), events (11.01), highly active antiretroviral therapy (9.36), antiretroviral therapy (8.41), death (8.41).
#9	17	Heart disease (20.57), HIV 1 infection (11.2), follow up (6.48), antiretroviral therapy heart (5.9), ambulatory blood pressure (5.9).
#10	17	HIV infection (38.78), chlamydia pneumoniae (8.59), plaque (8.29), adults (6.96), stents (6.15)..
#11	17	Human immunodeficiency virus (29.26), heart failure (18.34), diastolic dysfunction (17.04), vascular biology (11.36), cardiopulmonary bypass (11.36).
#12	13	Myocardial infarction (23.97), management (12.13), data collection (8.69), infection (6.87), acute myocardial infarction (6.33).

Similar to other bibliometric articles, there are 3 limitations of this study: the first one is that only the WoSCC database was used; the second one is the language was limited to English; the third one is article type was limited to the article, which may lead to missing data in some literature. In addition, some authors’ institutions and universities may have changed, resulting in the dispersion of research results, which may have affected the analysis results to some extent. Future research can further expand the database.

## 5. Conclusion

Based on 2 bibliometric analysis tools, CiteSpace and VOSviewer, this study explored the relationship between HIV/AIDS and CHD. We analyzed and summarized this research topic’s development process and hot trends. In recent years, HIV/AIDS has become a global public health problem, and CHD-related diseases are the critical factors in the death of PLHIV.^[48]^ If HIV infection factors, other effects of ART and traditional risk factors for CHD work together, PLHIV will face a higher risk of CHD infection. Therefore, future studies need to be more vigilant to reduce the risk of the combined effect of risk factors. This study lays the foundation for further research on the relationship between HIV/AIDS and CHD and future related research.

## Acknowledgments

We thank Peking University, The Sixth People Hospital of Zhengzhou, and ZaiShuiYiFang Smart Technology Limited Company for supporting our research.

## Author contributions

**Conceptualization:** Qiong Cai, Wei Pan, Ming Chun Zhang.

**Data curation:** Hui Xian Zhang, Yan Sun.

**Formal analysis:** Qiong Cai, Wei Pan.

**Funding acquisition:** Long Xi Pan.

**Methodology:** Qiong Cai.

**Project administration:** Jie Chun Wang, Yang Ming An.

**Software:** Qiong Cai.

**Validation:** Long Xi Pan.

**Visualization:** Fang Pan, Ping Jiang Xiao.

**Writing – original draft:** Qiong Cai, Wei Pan.

**Writing – review & editing:** Ming Chun Zhang, Long Xi Pan.
